# A purification strategy for analysis of the DNA/RNA-associated sub-proteome from chloroplasts of mustard cotyledons

**DOI:** 10.3389/fpls.2014.00557

**Published:** 2014-10-29

**Authors:** Yvonne Schröter, Sebastian Steiner, Wolfram Weisheit, Maria Mittag, Thomas Pfannschmidt

**Affiliations:** ^1^Lehrstuhl für Pflanzenphysiologie, Institut für Allgemeine Botanik und Pflanzenphysiologie, Friedrich-Schiller-Universität JenaJena, Germany; ^2^KWS SAAT AGEinbeck, Germany; ^3^Department of General Botany, Institute of General Botany and Plant Physiology, Friedrich Schiller University JenaJena, Germany; ^4^University of Grenoble-AlpesGrenoble, France; ^5^CNRS, UMR5168Grenoble, France; ^6^Commissariat a L'energie Atomique (CEA), iRTSV, Laboratoire de Physiologie Cellulaire & VégétaleGrenoble, France; ^7^INRA, USC1359Grenoble, France

**Keywords:** *Sinapis alba*, cotyledon, chloroplast, nucleic acids binding protein, post-translational modification, mass spectrometry

## Abstract

Plant cotyledons are a tissue that is particularly active in plastid gene expression in order to develop functional chloroplasts from pro-plastids, the plastid precursor stage in plant embryos. Cotyledons, therefore, represent a material being ideal for the study of composition, function and regulation of protein complexes involved in plastid gene expression. Here, we present a pilot study that uses heparin-Sepharose and phospho-cellulose chromatography in combination with isoelectric focussing and denaturing SDS gel electrophoresis (two-dimensional gel electrophoresis) for investigating the nucleic acids binding sub-proteome of mustard chloroplasts purified from cotyledons. We describe the technical requirements for a highly resolved biochemical purification of several hundreds of protein spots obtained from such samples. Subsequent mass spectrometry of peptides isolated out of cut spots that had been treated with trypsin identified 58 different proteins within 180 distinct spots. Our analyses indicate a high enrichment of proteins involved in transcription and translation and, in addition, the presence of massive post-translational modification of this plastid protein sub-fraction. The study provides an extended catalog of plastid proteins from mustard being involved in gene expression and its regulation and describes a suitable purification strategy for further analysis of low abundant gene expression related proteins.

## Introduction

Plant chloroplasts are semiautonomous cell organelles of endosymbiotic origin that emerged from a cyanobacteria-like ancestor (Lopez-Juez and Pyke, 2005). One evolutionary remnant of this origin is their own genome (called plastome) comprising 100–120 genes and a pre-dominantly bacteria-like gene-expression machinery being essential for its proper expression. The plastome gene set in vascular plants is highly conserved and encodes mainly proteins with a function in photosynthesis and the gene expression machinery (Sugiura, 1992). However, for full functionality plastids require the import of many proteins that are encoded by the nuclear compartment since during evolution the endosymbiotic ancestor lost most of its genes to the nucleus of the host cell *via* horizontal gene transfer (Martin et al., 2002; Stoebe and Maier, 2002). These nuclear-encoded plastid proteins are translated in the cytoplasm as precursor molecules that are subsequently imported into plastids with the help of N-terminal transit peptides directing them to their correct sub-compartment (Soll and Schleiff, 2004). After removal of the transit peptide the mature proteins are then assembled into their final configuration together with the plastid-expressed proteins and, therefore, all major multi-subunit complexes (such as photosystems, ribosomes, or metabolic enzyme complexes) represent a patchwork of nuclear as well as plastid expressed proteins (Allen et al., 2011).

Based on the prediction of transit peptides and genome-scale proteomics it was estimated that plastids may contain around 1500–4000 different proteins (Abdallah et al., 2000; Baerenfaller et al., 2008; Ferro et al., 2010; van Wijk and Baginsky, 2011). Reference proteomes generated for maize and *Arabidopsis* cover 1564 and 1559 proteins, respectively, so far (Huang et al., 2013) indicating that a large part of the predicted plastid proteome has yet not been detected. This might be caused by the fact that plastids from different tissues (for instance roots, cotyledons, leaves, flowers, and fruits) likely contain different protein compositions, but also from the fact that especially regulatory proteins are present in only trace amounts that are difficult to detect in a matrix of highly abundant proteins, e.g., from the photosynthetic apparatus (Huang et al., 2013). Further complexity in the plastid protein complement may derive from the occurrence of multiple post-translational modifications that are essential for regulatory events.

Cotyledons display a high activity in plastid transcription and translation being essential for the light-induced development of chloroplasts out of the embryonic pro-plastids (Baumgartner et al., 1989, 1993). Thus, the proteome of cotyledon plastids comprises a high amount of proteins implicated in gene expression providing a useful source material for the characterization of the nucleic acids binding proteome. The chloroplast proteome of the dicotyledonous model organism *Arabidopsis thaliana* is well studied in adult leaves, however, an analysis of that of cotyledons is lacking mainly because the small size of the cotyledons is not very suitable for the isolation of chloroplasts and subsequent analyses of their proteins *via* chromatography. In recent investigations, the fast growing cruciferous plant mustard (*Sinapis alba*) demonstrated a high suitability for performing biochemical and physiological analyses of plastid gene expression in cotyledons since the seedlings and their cotyledons are much larger than that of *Arabidopsis* (Oelmuller et al., 1986; Tiller and Link, 1993; Pfannschmidt and Link, 1994; Link, 1996; Baginsky et al., 1997). Isolation of cotyledons in the order of kilograms is easily achieved after just 5 days of growth and provides enough material even for the biochemical analysis of low-abundant proteins by chromatography followed by mass spectrometry. Since *Sinapis* is a close relative of *Arabidopsis*, peptide data evaluation for the identification of mustard plastid proteins was found to be applicable for well conserved proteins by using the *A. thaliana* or *Brassicales* protein databases (Schröter et al., 2010; Steiner et al., 2011). Thus, the use of mustard as a source for cotyledons combines the advantages of mustard chloroplast preparation with the availability of protein data of well studied organisms like *A. thaliana* or some *Brassica* species.

In recent studies, proteins implicated in plastid gene expression in mustard have been isolated by a number of different purification schemes. These include the isolation of the membrane bound insoluble transcriptionally active chromosome (TAC) by ultracentrifugation and gel filtration (Hallick et al., 1976; Bülow et al., 1987; Pfalz et al., 2006) and the isolation of soluble proteins such as RNA polymerases, kinases, RNA binding proteins and sigma factors by various chromatographic steps (Tiller et al., 1991; Nickelsen and Link, 1993; Tiller and Link, 1993; Pfannschmidt and Link, 1994; Liere and Link, 1995; Baginsky et al., 1999). Recently, we applied the purification scheme of plastid isolation followed by protein enrichment *via* heparin-Sepharose (HS) chromatography and visualization by two-dimensional (2D) blue native (BN)-PAGE to isolate protein complexes such as the RNA polymerase complex as well as a number of gene expression related proteins (Schröter et al., 2010; Steiner et al., 2011). However, these HS purified fractions still included a number of metabolic enzymes which exacerbate the analysis of the nucleic acids binding sub-proteome as they tend to cover low abundant proteins or even hinder their visualization and identification. Here, we present a pilot characterization of the nucleic acids binding sub-proteome of chloroplasts from mustard cotyledons. To this end we used HS chromatography followed by a second chromatographic step with phosphocellulose (PC) which was shown to be very effective for isolating nucleic acids binding enzymes like RNA polymerases (Bottomley et al., 1970; Tiller and Link, 1993). This was followed by isoelectric focussing (IF) and 2D gel electrophoresis that allowed us to estimate the size of the nucleic acids binding sub-proteome and the ideal IF range for its visualization and protein determination using mass spectrometry. The use of 2D gel electrophoresis also revealed massive post-translational modifications of the sub-proteome.

## Results

### Enrichment of nucleic acids binding proteins from mustard chloroplasts

**Figure 1:**
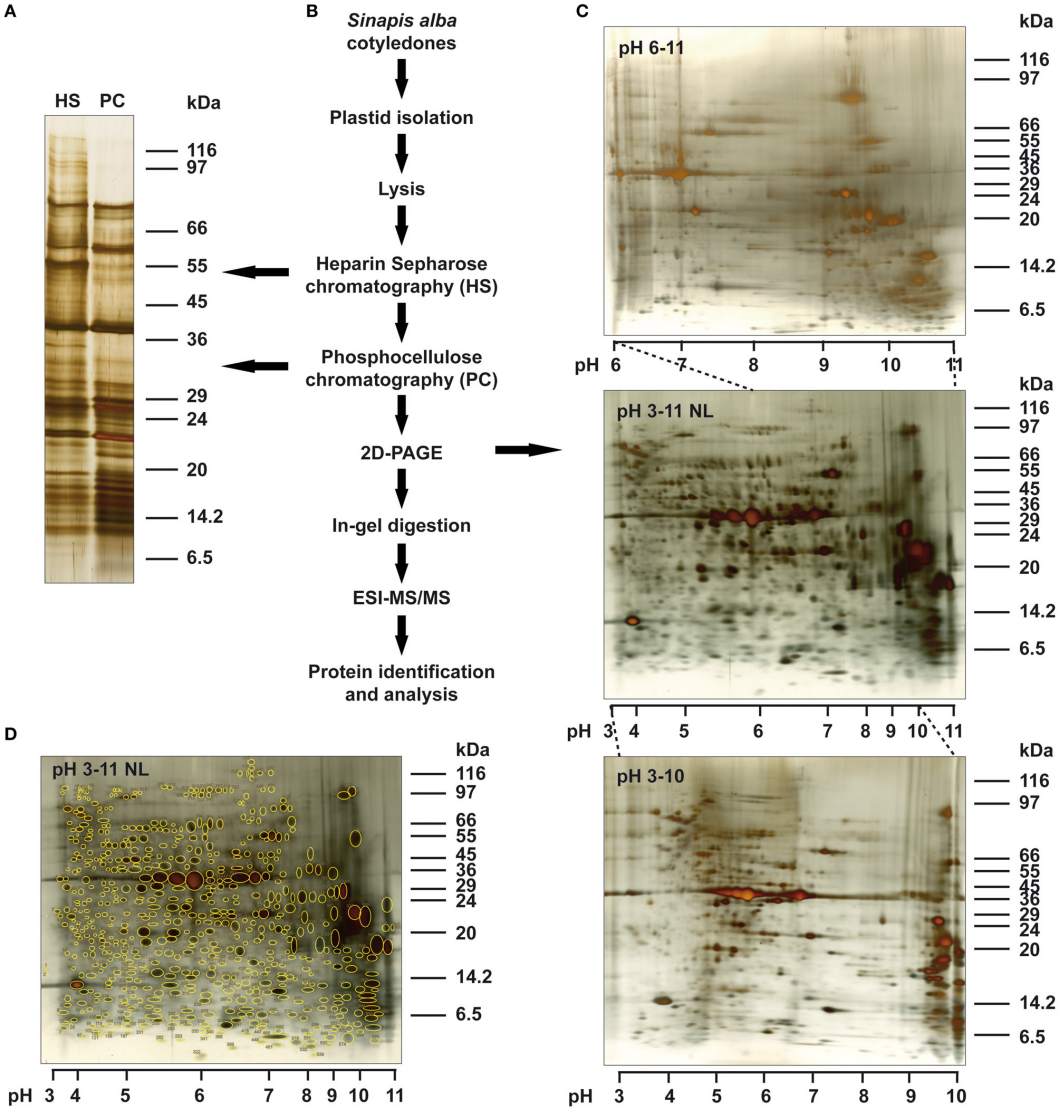
**Overview of purification procedure and protein visualization by 2D PAGE. (A)** Protein peak fractions after HS and PC chromatography in a silver stained 7–20% SDS polyacrylamide gel. Twenty microgram of protein per lane was separated. Sizes of marker proteins are given in the right margin. **(B)** Flow chart of the complete protein isolation and identification procedure. Arrows indicate the purification stages separated by 1D **(A)** or 2D PAGE **(C)**. **(C)** Protein pattern of PC peak fractions in silver stained 7.5–20% SDS acrylamide gels using three different pH gradients in the first dimension. The pH gradient used is indicated in the upper left corner of each gel and the pH range is given in detail below each gel. Marker sizes are given in the right margin. Dotted lines indicate the overlapping pH areas. Four hundred microgram of total protein separated in each gel. **(D)** Numbering of protein spots visualized in the 2D gel with pH 3–11NL for first dimension as shown in **(C)**. Marker sizes and pH range are given in the margin or below the gel, respectively.

In previous studies we analyzed gene expression related protein complexes from isolated mustard chloroplasts using a combination of HS chromatography followed by a two dimensional BN/SDS polyacrylamid gel-electrophoresis (2D BN-PAGE) and electro-spray ionization-tandem mass spectrometry (ESI-MS/MS). Besides the plastid-encoded RNA polymerase, various CSP41 complexes and translation related proteins, we identified several metabolic enzyme complexes such as GAP-dehydrogenase, ATPases, or RubisCO that co-purify in this affinity chromatography. These abundant proteins exacerbated the identification of further low-abundant proteins (Schröter et al., 2010; Steiner et al., 2011). In addition, these studies were focussed on the analysis of large native protein complexes using a BN-PAGE approach. This limited the characterization of gene expression related proteins that may occur in small complexes or as individual proteins. In this study, we aimed a deeper investigation of the size, composition and complexity of the nucleic acids binding subproteome of mustard chloroplasts. To this end, we performed chloroplast isolation and HS chromatography from mustard cotyledons precisely as described before (Schröter et al., 2010). Bound proteins were eluted with a high-salt step, concentrated by dialysis and, for further enrichment of gene expression related proteins, applied to a cation exchange column with PC as matrix as described earlier (see above). Proteins were eluted by a second high-salt step and dialyzed against a low-salt storage buffer for analysis and further use (see Materials and Methods). A first comparison of peak fractions with equal protein amounts of both purification steps was done by SDS-PAGE and silver staining (Figure 1A). The PC fraction exhibited a selective enrichment of many protein bands between 5 and 75 kDa and a strong exclusion of proteins larger than 75–80 kDa. For a more detailed resolution of this protein fraction, we performed 2D gel electrophoresis with an IF as first dimension followed by a SDS-PAGE (Figures 1B,C) as second dimension. Using IPG stripes with a non-linear (NL) pH range from 3 to 11 for the IF and a gradient polyacrylamide gel, we could obtain an overview of the total protein content leading to the identification of around 600 individual spots. We observed two major areas where multiple proteins accumulated on the gel which were located between approximately pH 4.5–7 and pH 9–11. Because of the non-linearity of the IF gradient, proteins at the outer ranges of the IF stripe were poorly resolved which became mainly evident at the basic pH values. Therefore, linear IPG gels were used in addition, overlapping with the first one between pH 3–10 and pH 6–11. The latter gradient resolved the problem with spot accumulation especially observed at the cathode. The higher resolution led to the identification of further proteins leading to a total count of 1079 individual protein spots within the PC fraction which could be distinguished between the different gels. We regard this as the nucleic acids binding sub-proteome of mustard plastids. Our data indicate a significant higher complexity of this specific sub-proteome as it was estimated earlier from the HS fractions (Schröter et al., 2010).

### Identification of proteins from the PC fraction by LC-ESI-MS/MS

**Figure 2:**
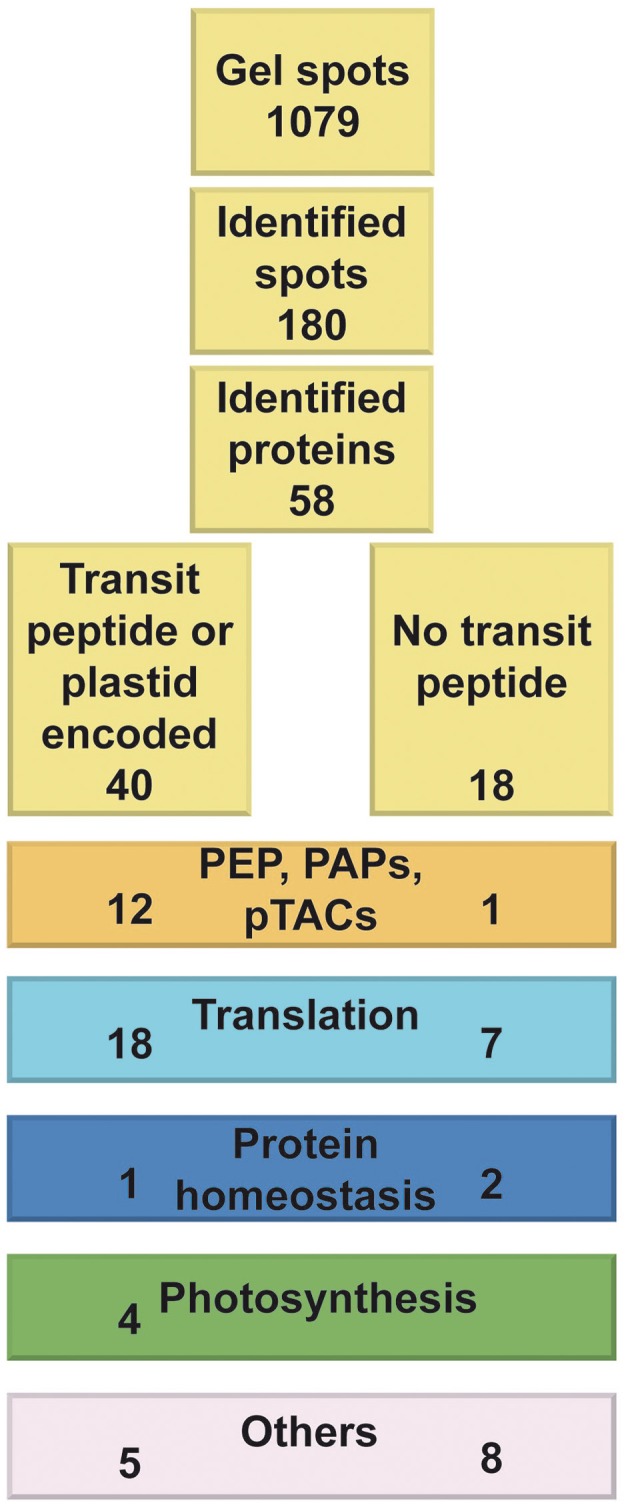
**Numbers of total, analyzed, and identified spots of the PC peak fractions**. Proteins separated in the 2D gels shown in Figure 1C are given in yellow boxes at the top. Protein groups corresponding to Table 1 are displayed below in colored boxes. At the left side of each box the number of putative plastid proteins per bin being either plastid encoded or for which a plastid transit peptide was predicted is given. At the right side proteins without these properties are given.

All 1079 spots were cut out and proteins were subjected to an in-gel tryptic digest. In 153 cases, selected spots were pooled from duplicate gels in order to increase the protein amount for the subsequent measurements. Since a database from *S. alba* is currently not available, protein identification was performed by comparing the determined mass spectrometry data to the *Brassicales* and *A. thaliana* databases (compare Materials and Methods). By this means 225 proteins were reliably identified with at least two different peptides in 180 spots indicating that several spots contained more than one protein. In addition, 36 particular proteins were identified in more than one spot (up to 40 different ones) suggesting post-translational modification of these proteins (Table 1). In total, 58 different proteins were identified. In further analyses, the identified gene models were checked for presence of a plastid transit peptide using TargetP (Emanuelsson et al., 2000) (Figure 2). Plastid-directing transit peptides could be predicted for 36 of these proteins, ten of them exhibit an additional luminal transit peptide and four plastid-encoded proteins were identified. Considering a detection probability of 73% for a transit peptide, we estimated the percentage of true plastid proteins within the PC fraction to be around 94%. Some of the identified proteins were found before in mustard (Pfannschmidt et al., 2000; Pfalz et al., 2006; Schröter et al., 2010), but 36 were identified here for the first time (Table 1).

**Table 1:** **Functional categorization and characterization of proteins from the phosphocellulose fraction identified by LC-ESI-MS/MS**.

**Protein**	**Spots**	**MapMan bin**	**Accession**	**ATG**	**cTP**	**References**
**PEP, PAPs, pTACs**						
Chloroplast RNA steem-loop binding protein 41a (CSP41a)	8	27.3.99	15229384	AT3G63140	cTP 0.983 RC 1	Pfannschmidt et al., 2000
Chloroplast RNA steem-loop binding protein 41b (CSP41b)	40	27	15217485	AT1G09340	–	Schröter et al., 2010
Fructokinase-like 1 (FLN1)	3	29.4.1	15232415	AT3G54090	cTP 0.624 RC 3	Pfalz et al., 2006
Iron superoxide dismutase 3 (FSD3)	1	21.6	15237281	AT5G23310	cTP 0.945 RC 2	Pfannschmidt et al., 2000
Plastid encoded RNA polymerase alpha (RpoA)	2	27.2	7525065	AtCg00740	–	Pfannschmidt et al., 2000
Plastid transcriptionally active chromosome 4 (pTAC4)	4	29.3.3	18408237	AT1G65260	cTP 0.965 RC 1	Pfalz et al., 2006
Plastid transcriptionally active chromosome 6 (pTAC6)	1	28.3	79318316	AT1G21600	cTP 0.802 RC 2	Pfalz et al., 2006
Plastid transcriptionally active chromosome 7 (pTAC7)	1	35.2	334187898	AT5G24314	cTP 0.899 RC 2	Pfalz et al., 2006
Plastid transcriptionally active chromosome 10 (pTAC10)	3	28.3	297816052	–	cTP 0.791 RC 3	Pfalz et al., 2006
Plastid transcriptionally active chromosome 12 (pTAC12)	1	28.3	30686151	AT2G34640	cTP 0.563 RC 4	Pfalz et al., 2006
Plastid transcriptionally active chromosome 18 (pTAC18)	1	35.2	15225202	AT2G32180	cTP 0.712 RC 4	Pfalz et al., 2006
Thioredoxin z (TRX z)	1	21.1	15230779	AT3G06730	cTP 0.858 RC 3	Pfalz et al., 2006
UDP-N-acetylmuamoylalanyl-d-glutamate-2,6-diaminopimelate ligase (MurE)	3	28.3	240254313	AT1G63680	cTP 0.695 RC 3	Pfalz et al., 2006
**TRANSLATION**						
Alpha-nascent polypeptide associated complex like protein 1 (Alpha-NAC-like protein 1)	1	29.2.4	15230476	AT3G12390	–	This work
Alpha-nascent polypeptide associated complex like protein 3 (Alpha-NAC-like protein 3)	1	29.2.4	240256288	AT5G13850	–	This work
Cytosolic ribosomal protein L11 (CRPL11)	1	29.2.1.2.2.11	79595462	AT2G42740	–	This work
Cytosolic ribosomal protein L22-2 (CRPL22-2)	2	29.2.1.2.2.22	145331980	AT3G05560	–	This work
Elongation factor 1-alpha4 (EF1-alpha4)	1	29.2.4	186532608	AT5G60390	–	This work
Elongation factor tu (EFtu)	1	29.2.4	15237059	AT4G20360	cTP 0.975 RC 1	Pfalz et al., 2006
Eukaryotic translation initiation factor 1A (eIF1A)	1	29.2.3	334188030	AT5G35680	–	This work
Eukaryotic translation initiation factor 3 (eIF3)	2	29.5.11.20	15225611	AT2G39990	cTP 0.797 RC 2	This work
Plastid ribosomal protein L1 (PRPL1)	21	29.2.1.1.1.2.1	15229443	AT3G63490	cTP 0.937 RC 1	This work
Plastid ribosomal protein L4 (PRPL4)	12	29.2.1.1.1.2.4	79317147	AT1G07320	cTP 0.826 RC 2	This work
Plastid ribosomal protein L5 (PRPL5)	1	29.2.1.1.1.2.5	15234136	AT4G01310	cTP 0.828 RC 3	This work
Plastid ribosomal protein L6 (PRPL6)	17	29.2.1.1.1.2.6	15220443	AT1G05190	cTP 0.495 RC 5	Schröter et al., 2010
Plastid ribosomal protein L10 (PRPL10)	6	29.2.1.1.1.2.10	15240644	AT5G13510	cTP 0.887 RC 2	This work
Plastid ribosomal protein L12-1 (PRPL12-1)	2	29.2.1.1.1.2.12	15232274	AT3G27830	cTP 0.941 RC 1	Pfalz et al., 2006
Plastid ribosomal protein L12-3 (PRPL12-3)	2	29.2.1.1.1.2.12	15232276	AT3G27850	cTP 0.955 RC 1	This work
Plastid ribosomal protein L14 (PRPL14)	1	29.2.1.1.1.2	297848252	–	–	This work
Plastid ribosomal protein L15 (PRPL15)	2	29.2.1.1.1.2.15	15230931	AT3G25920	cTP 0.866 RC 2	This work
Plastid ribosomal protein L18 (PRPL18)	1	29.2.1.1.1.2.18	15221153	AT1G48350	cTP 0.863 RC 2	This work
Plastid ribosomal protein L21 (PRPL21)	2	29.2.1.1.1.2.21	15219695	AT1G35680	cTP 0.981 RC 1	This work
Plastid ribosomal protein L24 (PRPL24)	2	29.2.1.1.1.2.24	30696487	AT5G54600	cTP 0.920 RC 2	This work
Plastid ribosomal protein L29 (PRPL29)	1	29.2.1.1.1.2.29	257717595	–	cTP 0.924 RC 2	Pfalz et al., 2006
Plastid ribosomal protein S5 (PRPS5)	14	29.2.1.1.1.1.5	15226167	AT2G33800	cTP 0.929 RC 1	This work
Translation initiation factor 2 (IF2)	7	29.2.3	15220055	AT1G17220	cTP 0.537 RC 3	This work
Translation initiation factor 3 (IF3)	2	29.2.3	18417644	AT4G30690	cTP 0.782 RC 2	This work
tRNA/rRNA methyltransferase (SpoU)	3	29.2.7	30680811	AT2G19870	–	This work
**PROTEIN HOMEOSTASIS**						
Chloroplast heat shock cognate protein 70-2 (cpHSC70-2)	1	29.6	15240578	AT5G49910	cTP 0.993 RC 1	This work
Protein disulfide isomerase like 2-1 (PDI-like 2-1)	2	21.1	145331431	AT2G47470	–	This work
T-complex protein 1/chaperonin60 family protein (TCP1/cpn60)	4	29.6	15242093	AT5G20890	–	This work
**PHOTOSYNTHESIS**						
ATPsynthase alpha	1	1.1.4	7525018	AtCg00120	–	Schröter et al., 2010
ATPsynthase beta	3	1.1.4	7525040	AtCg00480	–	Schröter et al., 2010
Rieske Cluster	2	1.1.3	30679426	AT4G03280	cTP 0.652 RC 3	This work
RubisCO activase	2	1.3.13	30687999	AT2G39730	cTP 0.888 RC 1	This work
**OTHERS**						
Acetyl-coenzyme A carboxylase carboxyl transferase subunit alpha (CAC3)	1	11.1.1	30687368	AT2G38040	cTP 0.927 RC 2	This work
Actin	1	31.1	79324605	AT2G37620	–	This work
Cruciferin 3 (CRU3)	2	33.1	15235321	AT4G28520	–	This work
Cystein synthase	1	13.1.5.3.1	334184908	AT2G43750	cTP 0.938 RC 1	This work
Fatty acid biosynthesis z (FabZ)	2	11.1.5	72255615	–	cTP 0.872 RC 2	This work
Malate dehydrogenase (MDH)	3	8.2.99	15232820	AT3G47520	cTP 0.911 RC 1	This work
Malate synthase (MLS)	4	6.2	334187411	AT5G03860	–	This work
Multi-functional protein 2 (MFP2)	5	11.9.4.9	15231317	AT3G06860	–	Schröter et al., 2010
Myrosinase	1	16.5.1	127734		–	This work
Phosphoserine aminotransferase (PSAT)	4	13.1.5.1.2	15237069	AT4G35630	cTP 0.938 RC 1	This work
Pyrroline-5-carboxylate reductase (P5CR)	3	13.2.2.3	145334418	AT5G14800	–	This work
Serine hydroxymethyltransferase 1 (SHMT 1)	8	25.1	15235745	AT4G37930	–	Schröter et al., 2010
Serine hydroxymethyltransferase 2 (SHMT 2)	8	25.1	30690404	AT5G26780	–	Schröter et al., 2010

*Identified proteins are named in the first column according to the annotation of the respective gene at NCBI. They are grouped into different classes written bold at the beginning of each group as defined in results. Proteins within each group are sorted alphabetically. Spots: number of the spots containing the respective protein. MapMan bin: classification groups for proteins according to the modified MapMan system of the plant proteome database (ppdb) [(http://ppdb.tc.cornell.edu/dbsearch/mapman.aspx) ^©^Klaas J. van Wijk Lab, Cornell University; Sun et al., 2009] based on the MapManBins of Thimm et al. (2004); Accession: gi identification number and At gene accession number; cTP: possibility of a plastid transit peptide and the respective reliability class (RC); References: first identification of the protein in mustard by mass spectrometry*.

**Figure 3:**
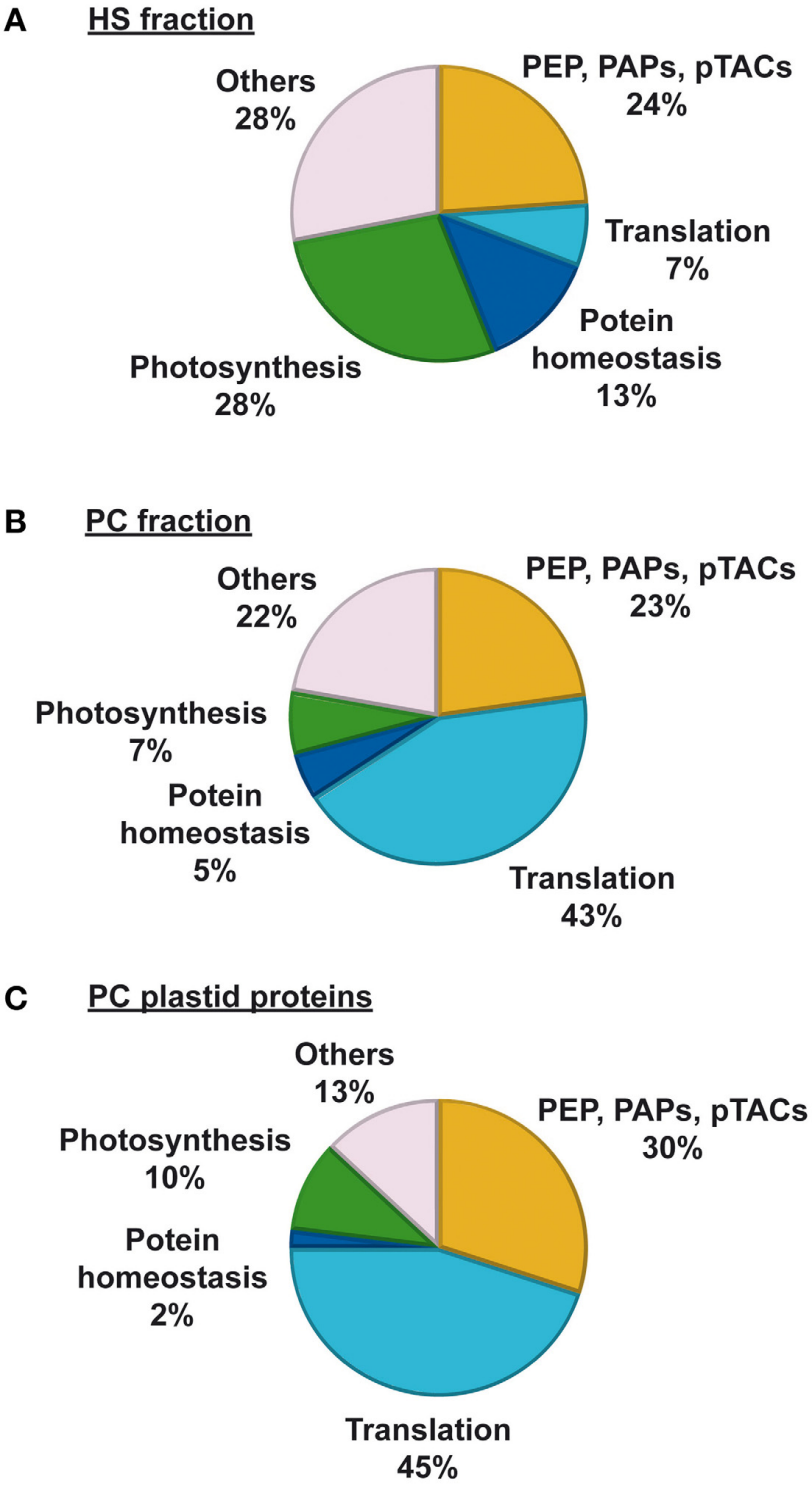
**Overview and comparison of the protein content in HS and PC fractions. (A)** Distribution of the identified proteins of the HS fractions analyzed in Schröter et al. (2010) and classification into groups in correlation to the recent work. **(B)** Percentage of identified proteins of PC peak fractions analyzed in this work and classified into groups as shown in Table 1. **(C)** Distribution of solely the plastid proteins of the recent PC fractions to functional groups according to Table 1 but with an aggregation of “PEPs and PAPs” with “Other pTACs” and a part of “DNA and RNA” to one bin “Transcription.”

Based on functional similarities and structural homologies, a categorization of proteins into protein families or subgroups was conducted (Figure 3). A practical classification mode is given by the modified MapMan bin system (Thimm et al., 2004) of the Plant Proteomics Data Base (PPDB) (Sun et al., 2009). In Table 1, proteins were listed following the PPDB bin grouping as given in column 2. For further comparison, we summarized identified proteins into five major groups. The first group comprises transcription and transcript related proteins, namely subunits of the plastid encoded RNA polymerase (PEPs) and PEP associated proteins (PAPs) as defined in Steiner et al. (2011), other pTACs (pTAC proteins not belonging to the PAPs) and RNA and DNA related proteins (bin 27 and 28, not belonging to PAPs and pTACs). A second large group comprises translation related proteins (bin 29.2 and 29.5). Three further groups cover proteins involved in protein homeostasis (bin 29 and 21 not belonging to PEPs and PAPs), photosynthesis (bin 1) and a miscellaneous group called “others” including various enzymes catalyzing metabolic reactions or protein modifications.

### PEPs, PAPs, and other pTACs

**Figure 4:**
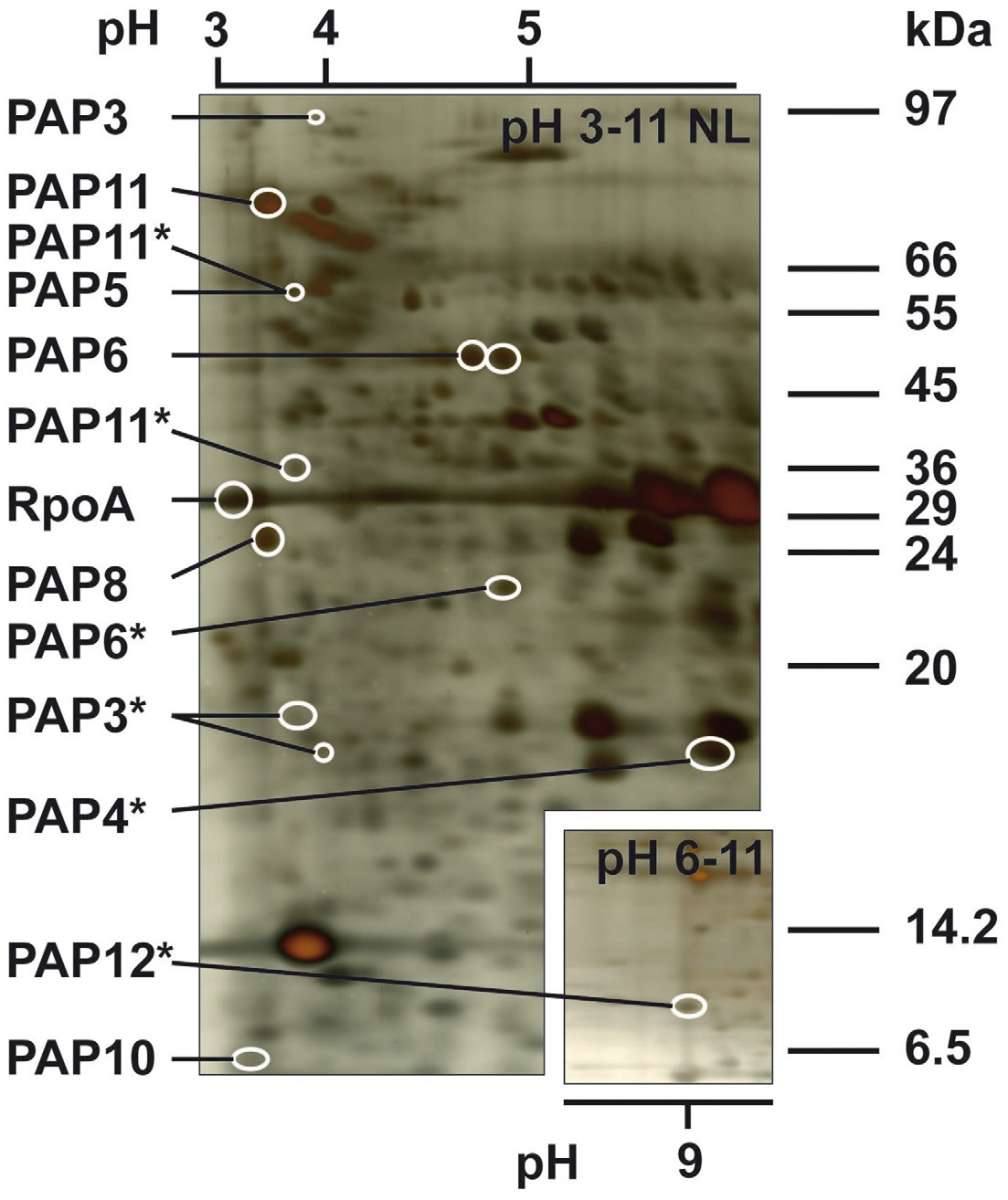
**Essential polymerase-associated proteins (PAPs) of the soluble PEP complex**. Positions of PAPs in the 2D gel after isoelectric focussing of the PC fraction on a pH 3–11NL and pH 6–11 gradient. Spot identity is given at the right margin. Fragments are additionally indicated by an asterisk. Marker sizes and pH range are given at right margin and above or below the gel, respectively. The gel is silver-stained.

We detected most subunits of the soluble PEP complex including PAP3, PAP4, PAP5, PAP6, PAP8, PAP10, PAP11, PAP12 as well as the PEP core subunit RpoA (Pfalz and Pfannschmidt, 2013). Other PEP core subunits (RpoB, RpoC1, RpoB) and PAP1, PAP2, PAP7, and PAP9 were not identifiable in spots of these gels. Most of the identified proteins of this group became visible as single isolated spots in the acidic range (pH 3–6) on the gel (Figure 4) and at their expected molecular weight. An exception was PAP6 representing the protein fructokinase-like 1 (FLN1) that contains a protein domain of the pfkB-carbohydrate kinase family (Arsova et al., 2010; Steiner et al., 2011). This protein appeared in a chain of five spots of the same apparent molecular weight but with slightly varying isoelectric points from which the two strongest spots were identified as PAP6 here. This observation suggests post-translational modification of this kinase. In addition, for PAP6 but also for PAP3 and PAP11 one or two spots of lower molecular weight, respectively, were detected suggesting a targeted degradation or proteolytic modification of these two proteins (Figure 4). For PAP4 and PAP12 only a degradation product was detectable, while a spot of the full length protein was not identified.

Besides PEP and PAP proteins, we identified two proteins described as component of the TAC in mustard, PTAC4 and PTAC18 (Pfalz et al., 2006). PTAC4 is the vesicle-inducing protein in plastids 1 (VIPP1) which plays a crucial role in membrane stability (Zhang et al., 2012). The PTAC18 protein belongs to the cupin superfamily that merges proteins with a conserved β-barrel fold, giving this type of protein a strong thermal stability. It represents a family of very diverse members including enzymes and seed storage proteins, but also transcription factors (Dunwell et al., 2001). However, the exact function of pTAC18 is largely unknown. PTAC18 was identified in spot 255 being smaller and more in the acidic range as expected from the predicted protein representing likely a fragment. PTAC4 was identified in spots 243, 278, 280, and 295. Two hundred and seventy-eight and 280 are on the same size but with slightly different IPs suggesting post-translational modification of the protein.

An exceptional constituent of the PC protein fraction represents the protein CSP41 that appears in two forms, CSP41a and CSP41b. Originally described as the chloroplast stem-loop binding protein of 41 kDa (Yang et al., 1996) it has been discussed to be involved in RNA processing and stabilization as well as in RNA protection (Qi et al., 2012). As described for the HS fractions it represents a dominant protein of the nucleic acids binding proteome of plastids being present in multiple multimeric complexes of highly variable sizes (Schröter et al., 2010; Qi et al., 2012). In the PC fractions, the two forms of CSP41 appear to be especially enriched as they can be detected in 10 spots of the same apparent molecular weight of around 34 kDa but with different IPs (three for CSP41a and seven for CSP41b). The main accumulation is visible in the middle of the gels between pH 5.5 and 7. The CSP41a spots are by far the strongest spots observed in the whole gel followed by the spots for CSP41b. Roughly estimated they account for 30–40% of the total protein content in this fraction making a precise estimate difficult. In addition, the proteins are detectable in 34 less stained and smaller spots of different sizes suggesting massive post-translational modifications as well as multiple degradation or targeted proteolytic events acting on both protein forms. These smaller protein spots of CSP41a/b appear to contain not only random fragments of the proteins but could be observed as reproducible spot pattern in all replicates of nucleic acids binding sub-proteome preparations from mustard.

### Translation associated proteins

Numerous proteins identified in this work are directly or indirectly related to translation. In total 12 ribosomal proteins of the large 50S subunit of plastid ribosomes (PRPL) were identified, namely PRPL1, -4, -5, -6, -10, -12, -14, -15, -18, -21,-24, -29. The solely identified protein of the small 30S ribosomal subunit (PRPS) is PRPS5. We also identified two ribosomal subunits that belong to the large subunit of the cytosolic 80S ribosomes (CRPL), CRPL11 and -22-2. *S. alba* proteins of PRPL12-1 and PRPL29 were formerly identified by Pfalz et al. (2006) and PRPL6 by Schröter et al. (2010). The remaining ribosomal proteins listed in Table 1 are identified in mustard plastid protein samples here for the first time.

Beside the ribosomal subunits a number of translation initiation factors (IF) were present in the fractions and were detected here for the first time in *S. alba*. Except of eIF1A (a subunit of the cytosolic translation initiation complex) all of them contain a predicted plastid transit peptide. This accounts also to eIF3 which is known as a subunit of a eukaryotic IF (eIF). IF2 and IF3 represent plastid translation IF while elongation factors (EF) EF-Tu and the eukaryotic EF1alpha4 are involved in translation elongation. eIF1A, EF-Tu, and EF1-alpha4 appear as single spots while the others were found in several spots suggesting post-translational modifications here, too.

Furthermore, we identified a SpoU methylase that belongs to the class of SPOUT enzymes and introduces a methylation of 2′-OH groups of tRNA or rRNA riboses (Cavaillé et al., 1999; Tkaczuk et al., 2007), and two proteins that are subunits of the nascent polypeptide associated complex (NAC). This dimeric complex is composed of an alpha- and beta-chain and may reversibly bind to ribosomes (Wiedmann et al., 1994). The alpha-NAC-like proteins identified during this work are encoded by different genes in *Arabidopsis* but exhibit a strong similarity within their amino acid sequence. The α-NAC like protein 1 and 3 were determined in the same two spots on the gels representing double spots.

### Proteins involved in protein homeostasis, photosynthesis, and metabolism

We identified the chloroplast heat shock cognate protein 70-2 (cpHsc70-2) which is the analog of one of only two stromal Hsp70s in *A. thaliana* plastids (Su and Li, 2008). In addition, we found a TCP-1/cpn60 family chaperonin and a protein disulfide isomerase like 2-1 (PDIL 2-1) belonging to the thioredoxin superfamily and acting as folding catalyst. All proteins are identified in mustard fractions here for the first time and likely function in protein stability or formation. The correct folding of proteins is the last but essential step of gene expression.

The group of photosynthesis related proteins contains four proteins. The alpha and beta subunits of the plastid ATP synthase were formerly identified in *S. alba* (Schröter et al., 2010). Another ATPase, the RubisCO activase and the Rieske cluster of the cytochrome b6/f complex were detected here first by mass spectrometry in the mustard plastid proteome. These proteins are most likely not involved in gene expression but co-purify in the column chromatography because of their substrate affinities. This is also true for the group of the miscellaneous proteins including the malate dehydrogenases (MDH) and the malate synthase (MLS), both identified in several spots.

Proteins involved in fatty acid metabolism were identified as well. These include acetyl-coenzyme A carboxylase carboxyl transferase subunit alpha (CAC3) and FabZ, a beta-hydroxyacyl-acyl carrierprotein (ACP) dehydratase. An earlier study on the purification of the acetyl-CoA carboxylase multienzyme complex also resulted in the enrichment of nucleoid-associated proteins (Phinney and Thelen, 2005) suggesting a potential physical link between these two larger protein associations.

A third protein found (MFP2) is involved in lipid degradation. It was already identified in the HS-fractions in former experiments (Schröter et al., 2010). We found also a cystein synthase and phosphoserine aminotransferase (PSAT) as well as a pyrroline-5-carboxylate reductase (P5CR) known to be essential for amino acid metabolism and a serine hydroxymethyltransferase (SHMT) being essential for photorespiration. The mustard protein in the PC fractions matches to mitochondrial SHMT1 and 2 peptides of several *Brassicales*. The exact affiliation to one of these SHMTs remains unclear since the matching peptides fit to both proteins (Table 1). The PC fractions contain also the myrosinase MB3 (involved in glucosinolate degradation) and a cruciferin fitting best to *A. thaliana* CRU3 (Table 1). Finally, also actin was detected in one spot, although mustard peptides of PC fractions match to different actin types of different *Brassicales*.

## Discussion

### The plastid nucleic acids binding proteome of mustard

Goal of our study was the establishment of a purification scheme allowing the estimation of size and composition of the plastid nucleic acids binding sub-proteome from mustard. By using HS and PC chromatography coupled to IF and SDS-PAGE we could reproducibly isolate 1079 protein spots from which we could identify 180 protein spots by mass spectrometry. However, to our surprise these 180 protein spots were found to represent just 58 individual proteins indicating a high degree of post-translational modification of this specific sub-proteome which in part might be caused by differential phosphorylation (Reiland et al., 2009, 2011). Since we used NaF as phosphatase inhibitor in all preparation steps, the differential phosphorylation states of the analyzed proteins should be well conserved. In contrast, different redox states of thiol groups were not maintained during our purification procedure since reducing agents were included in all steps. Detection of a differential redox state in these fractions will require more specific methods such as redox difference gel electrophoresis (redox-DIGE) (Hurd et al., 2007, 2009). We also observed numerous smaller fragments from several proteins indicating degradation events. These, however, were not random as the spot pattern was reproducible between different preparations suggesting that it is not caused by action of proteases during purification, but by targeted events in the chloroplast. Whether these products represent intermediate steps of protein degradation or whether these fragments perform distinct functions remains to be determined. In summary, this high degree of post-translational modification indicates that the size of the sub-proteome is certainly smaller than the 1079 spots detected. If we assume a similar percentage of individual proteins as within the identified spots (32.2%) for the complete fraction then we estimate 347 proteins for the total nucleic acids binding sub-proteome. Since we identified a number of co-purifying proteins involved in metabolic processes (29.3%), we had to reduce this number to 236 proteins. However, our mass spectrometry determination has a certain bias since we could detect only the fraction of sufficiently abundant proteins which likely is enriched in metabolic enzymes. In addition, a significant part of post-translational modification detected in our fractions is focussed on only two proteins, CSP41a and b which partly compromise our estimate. Without these two proteins, we estimate 314 proteins for the chloroplast nucleic acids binding sub-proteome. This appears a reasonable number taking into account the proteins that are already known to be involved in the regulation of plastid gene expression such as NEP, PEP, PAPs, pTACs, PPRs, ribosomal proteins and so on. It, however, leaves still some space for the discovery of as yet unidentified regulators that might appear only in trace amounts such as eukaryotic transcription factors (Wagner and Pfannschmidt, 2006).

### Specific features of the protein fraction after PC chromatography

PC chromatography is a well established purification step for nucleic acids binding proteins from chloroplasts (Bottomley et al., 1970; Tiller and Link, 1993). Crucial for the quality of these fractions, however, are a thorough chloroplast preparation *via* sucrose gradient centrifugation and a pre-purification step of the chloroplast lysate using HS chromatography. In comparison to results from earlier work using just HS fractions (Schröter et al., 2010) we observed a high enrichment of translation associated proteins and especially of CSP41 proteins. Co-purification of metabolic enzymes as well as components from other cell compartments was clearly reduced. We obtained a good coverage of the subunits for the plastid RNA polymerase complex PEP; however, surprisingly the larger subunits of this complex were not detectable. We observed a significant reduction of proteins above 80 kDa in size within the PC fractions (Figure 1), however, this might be not the reason for the failure of detection since all other components of the complex were identified in the fractions and especially RpoC2 and RpoB are known to bind DNA/RNA. Since these large subunits are highly conserved and have been successfully detected earlier in HS fractions (Steiner et al., 2011) it is likely that they are not well separated on the IEF. Further analyses using additional enrichment methodologies before the IEF step such as size-exclusion chromatography might help to target this problem in the future.

The largest amount of all identified proteins in the PC fractions is dedicated to translational processes with 43% of all proteins (Figure 3C). The 50S subunit of plastid ribosomes contains 33 subunits with 31 orthologs to *Escherichia coli* and the two plastid specific subunits PRPL5 and PRPL6 (Yamaguchi and Subramanian, 2000). The 30S subunit is composed of 21 *E. coli* orthologs and four plastid specific proteins with no homologs in other ribosomes (Yamaguchi et al., 2000). Most ribosomal proteins have contact to RNA in various ways, either they are structural components or directly involved in the translational process. Thus, ribosomal proteins contain nucleic acids binding structures which adhere to the used column materials and represent one main component of the nucleic acids binding sub-proteome of plastids. On the 2D-gels most of them accumulate at the higher pH-ranges and the use of the basic IPG-gels of pH 6–11 led to a good resolution of this group of proteins. The identification of 80S ribosomal proteins in plastid fractions is likely caused by the co-purification of particles attached to the outer chloroplast membrane, like known for tonoplast membrane fragments (Schröter et al., 2010). The main regulation of translation occurs at the level of initiation which is performed by initiation factors (IF). In eukaryotes this process is assured *via* 12 eIFs comprised by 23 polypeptides, whereas in prokaryotes three IFs are sufficient (Kapp and Lorsch, 2004). In plastids orthologs for all bacteria-type translation factors can be found, but the translational complex contains additional proteins not present in bacteria (Beligni et al., 2004). Three of the four IFs identified in this study contain a cTP although only IF2 and IF3 are plastid IFs with a prokaryotic origin. The third one, eIF3f, is a subunit of the eIF3 and is important for the basic cell growth and development and influences the expression of about 3000 genes in *A. thaliana* also in interaction with two other eIF3 subunits (Xia et al., 2010). ChloroP predicts a plastid transit peptide of 40 amino acids for eIF3f of *A. thaliana* and it was previously also identified in fractions enriched in plastid nucleoids (Huang et al., 2013). Thus, it seems to be a true plastid protein and not a co-purification of the cytosolic translational apparatus. However, it might be also possible that this protein possesses a dual localization both in nucleus and plastids contributing to the coordination of gene expression between the two genetic compartments as proposed for other plant cell proteins (Krause and Krupinska, 2009). The elucidation of the precise role of eIF3f in plastids and whether it is involved in the regulation of plastid gene expression will be an interesting field of future research.

**Figure 5:**
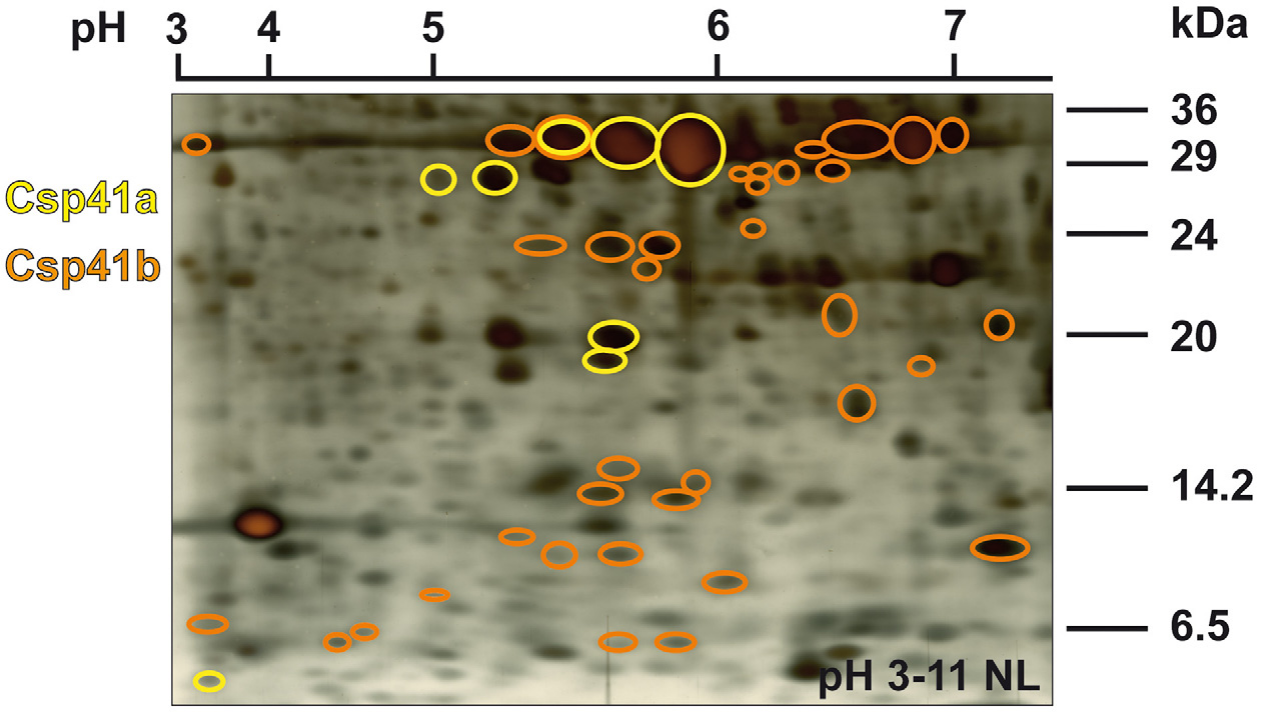
**Distribution of CSP41a and b spots in the pH 3–11 NL 2D-gel**. CSP41a is drawn in yellow and CSP41b in orange. Marker sizes and pH range are given right beside and above the silver stained gel, respectively.

The dominant proteins in the PC fractions are the two proteins named CSP41a and CSP41b (Yang et al., 1996; Yang and Stern, 1997). CSP41a and b were also detected in isolates of the PEP-complex as one of the most abundant component (Pfannschmidt et al., 2000; Suzuki et al., 2004; Schröter et al., 2010) but they appear not to belong to the PAPs but co-purify with these fractions because of the enormous size of their largest conglomerates (Peltier et al., 2006; Schröter et al., 2010; Qi et al., 2012). Here, we identified CSP41a in 8 and CSP41b in 40 spots of diverse sizes and isoelectric points. Thereby, both form a defined spot pattern which was congruent in most replicates of the 2D-gels prepared for this work. This suggests that not only a multimerization of CSP41a/b occurs but maybe also an integration of defined fragment species of the proteins that might be important for specific functions. In addition to targeted fragmentation, the spot pattern after 2D SDS-PAGE suggests also a strong post-translational modification of the two proteins. Indeed, phosphorylation and lysine acetylation have been reported for the corresponding *Arabidopsis* proteins (Reiland et al., 2009, 2011; Finkemeier et al., 2011). The spot pattern as well as the positions of the two proteins in the 2d-gels is highly reminiscent to those recently reported for *Arabidopsis* (Qi et al., 2012). The only difference occurs in the number of identified spots which were 6 Csp41a and 5 Csp41b in *Arabidopsis* while in mustard we observed 3 Csp41a and 7 Csp41b variants (besides the fragmented versions) (Figure 5). This suggests the action of at least some species-specific modifications of the proteins.

## Conclusion

Here, we describe the technical requirements for a highly resolved biochemical purification of several hundreds of protein spots representing the nucleic acids binding sub-proteome of plastids. Our analyses indicate a high enrichment of proteins involved in transcription and translation and, in addition, the presence of massive post-translational modification of this plastid protein sub-fraction. Furthermore, our study provides an extended catalog of plastid proteins from mustard being involved in gene expression and its regulation and describes a suitable purification strategy for further analysis of low abundant gene expression related proteins.

## Materials and methods

### Plant growth and isolation of plastids

Mustard seedlings (*Sinapis alba* L., var. Albatros) were cultivated under permanent white light illumination at 20°C and 60% humidity. Cotyledons were harvested under the respective light and stored on ice before homogenization in ice-cold isolation buffer in a Waring Blender and filtering through muslin and nylon. Chloroplast isolation by differential centrifugation and sucrose gradient centrifugation in a gradient between 30 and 55% sucrose was conducted as described earlier (Schröter et al., 2010).

### Isolation of nucleic acids binding proteins by HS- and PC-chromatography

Lysis of plastids and the chromatography at HS CL-6B was performed according to (Tiller and Link, 1993; Steiner et al., 2009). Proteins were washed, eluted with 1.2 M (NH_4_)_2_SO_4_ and the peak fractions detected via protein quantification assays (RC DC™, Bio-Rad Laboratories, Inc., Hercules, CA, USA) (Schröter et al., 2010). For PC chromatography pooled HS peak fractions were diluted to 10% (v/v) glycerol with dilution buffer [50 mM Tris/HCl, pH 7.6, 0.1 mM EDTA, 0.1% (v/v) TritonX-100, 10 mM sodium fluoride, 62.5 mM (NH_4_)_2_SO_4_, 6 mM 2-mercaptoethanol]. Activation and equilibration of PC (cellulose phosphate ion-exchanger P11, Whatman™ GE healthcare UK Limited, Little Chalfont, UK) to pH 7.6 occurred following the distributor's instructions. Diluted HS proteins were applied to disposable PD-10 columns (Amersham™ GE healthcare UK Limited, Little Chalfont, UK) filled with activated PC, closed carefully and rotated gently for 60 min at 4°C. After fixing the column on a stand and washing with washing buffer [50 mM Tris/HCl, pH 7.6, 0.1 mM EDTA, 0.1% (v/v) TritonX-100, 10 mM sodium fluoride, 50 mM (NH_4_)_2_SO_4_, 5 mM 2-mercaptoethanol, 10% (v/v) glycerol] proteins were eluted in 3 ml fractions with elution buffer [50 mM Tris/HCl, pH 7.6, 0.1 mM EDTA, 0.1% (v/v) TritonX-100, 10 mM sodium fluoride, 1.2 M (NH_4_)_2_SO_4_, 5 mM 2-mercaptoethanol, 10% (v/v) glycerol] and dialyzed against storage buffer [50 mM Tris/HCl, pH 7.6, 0.1 mM EDTA, 0.1% (v/v) TritonX-100, 10 mM sodium fluoride, 50 mM (NH_4_)_2_SO_4_, 5 mM 2-mercaptoethanol, 50% (v/v) glycerol]. Peak fractions were determined by a protein quantification assay (RC DC™, Bio-Rad Laboratories, Inc., Hercules, CA, USA), pooled and stored at −20°C.

### 2D gel electrophoresis

For 2D gel electrophoresis an acetone precipitation of dialyzed proteins from PC chromatography was used to remove the storage buffer following manuals instruction (2-D Electrophoresis principles and methods, 2004, GE healthcare UK Limited, Little Chalfont, UK). For first dimension 18 cm IPG-stripes pH 3–11NL, pH 6–11, and pH3–10 were used (GE Healthcare UK Limited, Little Chalfont, UK). IPG-stripes were rehydrated in rehydration buffer [8 M urea, 0.5% (w/v) chaps, 0.2% (w/v) DTT, 0.5% (v/v) IPG-Buffer, 0.002% (w/v) bromophenol blue] for about 14–16 h. An amount of 400 μg precipitated and dried protein per stripe was resolved in rehydration buffer and applicated on the IPG-stripes as cup-loading procedure following manufacturer's instructions (2-D Electrophoresis principles and methods, 2004, GE Healthcare UK Limited, Little Chalfont, UK). For focussing of proteins the following protocol was used on IPGphor (Amersham™ GE Healthcare UK Limited, Buckinghamshire, UK) 6 h step and hold 150 V, 3 h step and hold 300 V, 6 h gradient 1200 V, 3 h gradient 8000 V, 3 h step and hold 8000 V. After IF IPG-strips were equilibrated twice in equilibration solution [50 mM Tris-HCl, ph 8.8, 6 M urea, 30% (v/v) glycerol, 2% (w/v) SDS, 0.002% (w/v) bromophenol blue] first with addition of 2% (w/v) DTT for 15 min under gentle agitation and after removing the first solution second with addition of 2.5% (w/v) iodacetamide (IAA, for alkylation of thiol groups) again for 15 min and gently agitated as described (2-D Electrophoresis principles and methods, 2004, GE Healthcare UK Limited, Little Chalfont, UK). As second dimension a SDS PAGE in gradient gels of 7.5–20% acrylamide with Rhinohide™ gel strengthener (Molecular Probes, Inc., Eugene, OR, USA) was used following manual instructions. Afterwards gels were stained with silver according to manufacturer's instruction (Amersham™ GE Healthcare UK Limited, Buckinghamshire, UK).

### Tryptic digest, LC/ESI-MS/MS and data analysis

The spot pattern of the different gels was compared. Matching low abundant spots were pooled (as indicated in Supplemental Table 1) to increase the detectable protein amount. Tryptic digest of protein spots was conducted after destaining as referred (Mørtz et al., 1994; Stauber et al., 2003). Mass spectrometry was carried out at LCQ™-DecaXP ion trap mass spectrometer (Thermo Finnigan, San Jose, CA, USA) using a data-dependent scan procedure with four cyclic scan events as described in Schröter et al. (2010). The first cycle, a full MS scan of the mass range m/z 450–1200, was followed by three dependent MS/MS scans of the three most abundant ions. Sample run and data acquisition was performed using the Xcalibur™ software (Version1.3 ^©^ Thermo Finnigan 1998–2001). Seventy-six of the low abundant spots were measured at a Finnigan LTQ linear ion trap mass spectrometer (Thermo Finnigan, Thermo Fisher Scientific Inc., Waltham, MA, USA) coupled online after a nano HPLC Ultimate 3000 (Dionex, Thermo Fisher Scientific Inc., Waltham, MA, USA) (Schmidt et al., 2006). After one full MS the instrument was set to measure the collision induced dissociation pattern of the four most abundant ions and exclude the measured once for 10 s from newly measuring.

The resulting spectra were analyzed using the Proteome Discoverer vs. 1.0 (Thermo Fisher Scientific Inc., Waltham, MA, USA) with the implemented Sequest algorithm (Link et al., 1999). Therefore, a database of all RefSeq (reference sequence) sequences of *A. thaliana* and *Arabidopsis lyrata* as well as the complete *Brassica napus* and *Capsella rubella* and the remaining *Brassicales* proteins of NCBI was created [NCBI 2012.03.19 109146 sequences: *Arabidopsis* RefSeq 67924 sequences (35375 *A. thaliana*, 32549 *A. lyrata*) + *B. napus* 10622 sequences + *C. rubella* 4246 sequences + other *brassicales* 26354 sequences]. The Proteome Discoverer Software was set to adjust the Xcorr to reach a false discovery rate of ≤ 1% (Veith et al., 2009). All proteins with at least two unique peptides were taken for further analysis.

For transit peptide prediction the web-based tools TargetP (http://www.cbs.dtu.dk/services/TargetP/) (Emanuelsson et al., 2000) was used and for prediction of the transit peptide length the web-tool ChloroP (http://www.cbs.dtu.dk/services/ChloroP/) (Emanuelsson et al., 1999). For further analyses identified proteins were grouped into bins according to the modified MapMan system of the plant proteome database (ppdb) (http://ppdb.tc.cornell.edu/dbsearch/mapman.aspx) (Sun et al., 2009) based on the MapManBins of Thimm et al. (2004) (Supplemental Table 2).

### Conflict of interest statement

The authors declare that the research was conducted in the absence of any commercial or financial relationships that could be construed as a potential conflict of interest.

## Supplementary material

Supplemental Figure S1 **Silver stained 2D-gels of the PC fractions with isoelectric focusing for the first dimension in pH gradients between 6–11, 3–11 NL, and 3–10 indicated in the upper left corner of each gel**. The second dimension is performed in a 7.5–20% SDS polyacrylamide gel. Spots are marked and numbered in yellow. Marker sizes and pH range are given right beside and below the gel, respectively.

Supplemental Table 1 **Identified peptides from the PC fraction**. Spots are listed in numerical order. Accession numbers of proteins belonging to the same spot are listed in an order starting with the highest peptide coverage. Spot nr., identification number of the protein containing spot on the 2D-gels (see Supplemental Figure S1). Descriptions of depicted proteins are given as stated in the databases (see Materials and Methods). Coverage, coverage of the depicted proteins by the identified peptides; calc. pI, calculated pI of the depicted proteins based on the protein sequences in the database; MW, calculated molecular weight based on the protein sequences in the databases; z, peptide ion charge; lower case “m” in the peptide sequence, oxidized form of methionine; lower case “w” oxidized form of tryptophane; lower case “c” cystein with carbamidomethylation; lower case “k” acetylation of lysine.

Supplemental Table 2 **Detailed characterization of proteins from the PC fraction identified by LC-ESI-MS/MS**. Identified proteins are given in the first column according to the annotation of the respective gene at NCBI. Proteins were sorted according to the MapMan bin numbering in the second column representing classification groups for proteins according to the modified MapMan system of the plant proteome database (ppdb) [(http://ppdb.tc.cornell.edu/dbsearch/mapman.aspx) ^©^Klaas J. van Wijk Lab, Cornell University (Sun et al., 2009) based on the MapManBins of Thimm et al. (2004)]. Spots: number of spots containing the respective protein. Spot nr: identification number of the protein containing spot on the 2D-gels. NCBI accession: gi identification number at NCBI (The National Center for Biotechnology Information http://www.ncbi.nlm.nih.gov/); ATG: gene accession of the first matching Arabidopsis thaliana hit or (if no A. thaliana protein was matching) the best matching other organism and the respective A. thaliana gene accession determined by a protein-protein blast at NCBI in brackets; cTP: probability of a plastid transit peptide; MW [kDa]: calculated theoretical molecular weight in kilodalton; MW(-cTP) [kDa]: calculated theoretical molecular weight without chloroplast transit peptide (cTP) in kilodalton; MW(-lTP) [kDa]: calculated theoretical molecular weight without cTP and luminal transit peptide (lTP) in kilodalton; PI: calculated isoelectric point; PI (-cTP): calculated isoelectric point without cTP; PI (-lTP): calculated isoelectric point without cTP and lTP. The last six parameter were provided at the related accession entry by ppdb (http://ppdb.tc.cornell.edu/dbsearch/searchacc.aspx).

## References

[B1] Abdallah F. Salamini F. Leister D. (2000). A prediction of the size and evolutionary origin of the proteome of chloroplasts of Arabidopsis. Trends Plant Sci. 5, 141–142. 10.1016/S1360-1385(00)01574-0 10928822

[B2] Allen J. F. de Paula W. B. Puthiyaveetil S. Nield J. (2011). A structural phylogenetic map for chloroplast photosynthesis. Trends Plant Sci. 16, 645–655. 10.1016/j.tplants.2011.10.004 22093371

[B3] Arsova B. Hoja U. Wimmelbacher M. Greiner E. Ustun S. Melzer M.. (2010). Plastidial thioredoxin z interacts with two fructokinase-like proteins in a thiol-dependent manner: evidence for an essential role in chloroplast development in arabidopsis and *Nicotiana benthamiana*. Plant Cell 22, 1498–1515. 10.1105/tpc.109.071001 20511297 PMC2899873

[B4] Baerenfaller K. Grossmann J. Grobei M. A. Hull R. Hirsch-Hoffmann M. Yalovsky S.. (2008). Genome-scale proteomics reveals *Arabidopsis thaliana* gene models and proteome dynamics. Science 320, 938–941. 10.1126/science.1157956 18436743

[B5] Baginsky S. Tiller K. Link G. (1997). Transcription factor phosphorylation by a protein kinase associated with chloroplast RNA polymerase from mustard (*Sinapis alba*). Plant Mol. Biol. 34, 181–189. 10.1023/A:1005802909902 9207834

[B6] Baginsky S. Tiller K. Pfannschmidt T. Link G. (1999). PTK, the chloroplast RNA polymerase-associated protein kinase from mustard (*Sinapis alba*), mediates redox control of plastid *in vitro* transcription. Plant Mol. Biol. 39, 1013–1023. 10.1023/A:1006177807844 10344206

[B7] Baumgartner B. J. Rapp J. C. Mullet J. E. (1989). Plastid transcription and DNA copy number increase early in barley chloroplast development. Plant Physiol. 89, 1011–1018. 10.1104/pp.89.3.1011 16666609 PMC1055959

[B8] Baumgartner B. J. Rapp J. C. Mullet J. E. (1993). Plastid genes encoding the transcription/translation apparatus are differentially transcribed early in barley (*Hordeum vulgare*) chloroplast development. Plant Physiol. 101, 781–791. 12231729 10.1104/pp.101.3.781 PMC158691

[B9] Beligni M. V. Yamaguchi K. Mayfield S. P. (2004). The translational apparatus of *Chlamydomonas reinhardtii* chloroplast. Photosynth. Res. 82, 315–325. 10.1007/s11120-004-2440-5 16143843

[B10] Bottomley W. Smith H. J. Bogorad L. (1970). RNA polymerases of maize: partial purification and properties of the chloroplast enzyme. Proc. Natl. Acad. Sci. U.S.A. 71, 2412–2416. 5002819 10.1073/pnas.68.10.2412 PMC389433

[B11] Bülow S. Reiss T. Link G. (1987). DNA-binding proteins of the transcriptionally active chromosome from mustard (*Sinapis alba L*.) chloroplasts. Curr. Genet. 12, 157–159. 10.1007/BF00434671

[B12] Cavaillé J. Chetouani F. Bachellerie J. P. (1999). The yeast *Saccharomyces cerevisiae* YDL112w ORF encodes the putative 2′-O-ribose methyltransferase catalyzing the formation of Gm18 in tRNAs. RNA 5, 66–81. 10.1017/S1355838299981475 9917067 PMC1369740

[B13] Dunwell J. M. Culham A. Carter C. E. Sosa-Aguirre C. R. Goodenough P. W. (2001). Evolution of functional diversity in the cupin superfamily. Trends Biochem. Sci. 26, 740–746. 10.1016/S0968-0004(01)01981-8 11738598

[B14] Emanuelsson O. Nielsen H. Brunak S. von Heijne G. (2000). Predicting subcellular localization of proteins based on their N-terminal amino acid sequence. J. Mol. Biol. 300, 1005–1016. 10.1006/jmbi.2000.3903 10891285

[B15] Emanuelsson O. Nielsen H. von Heijne G. (1999). ChloroP, a neural network-based method for predicting chloroplast transit peptides and their cleavage sites. Protein Sci. 8, 978–984. 10.1110/ps.8.5.978 10338008 PMC2144330

[B16] Ferro M. Brugière S. Salvi D. Seigneurin-Berny D. Court M. Moyet L.. (2010). AT_CHLORO, a comprehensive chloroplast proteome database with subplastidial localization and curated information on envelope proteins. Mol. Cell. Proteomics 9, 1063–1084. 10.1074/mcp.M900325-MCP200 20061580 PMC2877971

[B17] Finkemeier I. Laxa M. Miguet L. Howden A. J. Sweetlove L. J. (2011). Proteins of diverse function and subcellular location are lysine acetylated in Arabidopsis. Plant Physiol. 155, 1779–1790. 10.1104/pp.110.171595 21311031 PMC3091095

[B18] Hallick R. B. Lipper C. Richards O. C. Rutter W. J. (1976). Isolation of a transcriptionally active chromosome from chloroplasts of *Euglena gracilis*. Biochemistry 15, 3039–3043. 10.1021/bi00659a016 821516

[B19] Huang M. Friso G. Nishimura K. Qu X. Olinares P. D. Majeran W.. (2013). Construction of plastid reference proteomes for maize and Arabidopsis and evaluation of their orthologous relationships; the concept of orthoproteomics. J. Proteome Res. 12, 491–504. 10.1021/pr300952g 23198870

[B20] Hurd T. R. James A. M. Lilley K. S. Murphy M. P. (2009). Measuring redox changes to mitochondrial protein thiols with redox difference gel electrophoresis (redox-DIGE). Methods Enzymol. 456, 343–361. 10.1016/S0076-6879(08)04419-4 19348898

[B21] Hurd T. R. Prime T. A. Harbour M. E. Lilley K. S. Murphy M. P. (2007). Detection of reactive oxygen species-sensitive thiol proteins by redox difference gel electrophoresis: implications for mitochondrial redox signaling. J. Biol. Chem. 272, 22040–22051. 10.1074/jbc.M703591200 17525152

[B22] Kapp L. D. Lorsch J. R. (2004). The molecular mechanics of eukaryotic translation. Annu. Rev. Biochem. 73, 657–704. 10.1146/annurev.biochem.73.030403.080419 15189156

[B23] Krause K. Krupinska K. (2009). Nuclear regulators with a second home in organelles. Trends Plant Sci. 14, 194–199. 10.1016/j.tplants.2009.01.005 19285907

[B24] Liere K. Link G. (1995). RNA-binding activity of the matK protein encoded by the chloroplast trnK intron from mustard (*Sinapis alba L*.). Nucleic Acids Res. 23, 917–921. 10.1093/nar/23.6.917 7537369 PMC306785

[B25] Link A. J. Eng J. Schieltz D. M. Carmack E. Mize G. J. Morris D. R.. (1999). Direct analysis of protein complexes using mass spectrometry. Nat. Biotechnol. 17, 676–682. 10.1038/10890 10404161

[B26] Link G. (1996). Green life: control of chloroplast gene transcription. Bioessays 18, 465–471. 10.1002/bies.950180608

[B27] Lopez-Juez E. Pyke K. A. (2005). Plastids unleashed: their development and their integration in plant development. Int. J. Dev. Biol. 49, 557–577. 10.1387/ijdb.051997el 16096965

[B28] Martin W. Rujan T. Richly E. Hansen A. Cornelsen S. Lins T.. (2002). Evolutionary analysis of Arabidopsis, cyanobacterial, and chloroplast genomes reveals plastid phylogeny and thousands of cyanobacterial genes in the nucleus. Proc. Natl. Acad. Sci. U.S.A. 99, 12246–12251. 10.1073/pnas.182432999 12218172 PMC129430

[B29] Mørtz E. Vorm O. Mann M. Roepstorff P. (1994). Identification of proteins in polyacrylamide gels by mass spectrometric peptide mapping combined with database search. Biol. Mass Spectrom. 23, 249–261. 10.1002/bms.1200230503 8204681

[B30] Nickelsen J. Link G. (1993). The 54 kDa RNA-binding protein from mustard chloroplasts mediates endonucleolytic transcript 3′ end formation *in vitro*. Plant J. 3, 537–544. 10.1046/j.1365-313X.1993.03040537.x 8220460

[B31] Oelmuller R. Dietrich G. Link G. Mohr H. (1986). Regulatory factors involved in gene-expression (subunits of ribulose-1,5-bisphosphate carboxylase) in mustard (*Sinapis-alba L*.) cotyledons. Planta 169, 260–266. 10.1007/BF00392323 24232559

[B32] Peltier J. B. Cai Y. Sun Q. Zabrouskov V. Giacomelli L. Rudella A.. (2006). The oligomeric stromal proteome of *Arabidopsis thaliana* chloroplasts. Mol. Cell. Proteomics 5, 114–133. 10.1074/mcp.M500180-MCP200 16207701

[B33] Pfalz J. Liere K. Kandlbinder A. Dietz K. J. Oelmuller R. (2006). PTAC2,-6, and-12 are components of the transcriptionally active plastid chromosome that are required for plastid gene expression. Plant Cell 18, 176–197. 10.1105/tpc.105.036392 16326926 PMC1323492

[B34] Pfalz J. Pfannschmidt T. (2013). Essential nucleoid proteins in early chloroplast development. Trends Plant Sci. 18, 186–194. 10.1016/j.tplants.2012.11.003 23246438

[B35] Pfannschmidt T. Link G. (1994). Separation of 2 classes of plastid DNA-dependent Rna-polymerases that are differentially expressed in mustard (*Sinapis-alba L*) Seedlings. Plant Mol. Biol. 25, 69–81. 10.1007/BF00024199 8003698

[B36] Pfannschmidt T. Ogrzewalla K. Baginsky S. Sickmann A. Meyer H. E. Link G. (2000). The multisubunit chloroplast RNA polymerase A from mustard (*Sinapis alba L*.) - integration of a prokaryotic core into a larger complex with organelle-specific functions. Eur. J. Biochem. 267, 253–261. 10.1046/j.1432-1327.2000.00991.x 10601874

[B37] Phinney B. S. Thelen J. J. (2005). Proteomic characterization of a Triton-insoluble fraction from chloroplasts defines a novel group of proteins associated with macromolecular structures. J. Proteome Res. 4, 497–506. 10.1021/pr049791k 15822927

[B38] Qi Y. Armbruster U. Schmitz-Linneweber C. Delannoy E. de Longevialle A. F. Rühle T.. (2012). Arabidopsis CSP41 proteins form multimeric complexes that bind and stabilize distinct plastid transcripts. J. Exp. Bot. 63, 1251–1270. 10.1093/jxb/err347 22090436 PMC3276088

[B39] Reiland S. Finazzi G. Endler A. Willig A. Baerenfaller K. Grossmann J.. (2011). Comparative phosphoproteome profiling reveals a function of the STN8 kinase in fine-tuning of cyclic electron flow (CEF). Proc. Natl. Acad. Sci. U.S.A. 108, 12955–12960. 10.1073/pnas.1104734108 21768351 PMC3150903

[B40] Reiland S. Messerli G. Baerenfaller K. Gerrits B. Endler A. Grossmann J.. (2009). Large-scale Arabidopsis phosphoproteome profiling reveals novel chloroplast kinase substrates and phosphorylation networks. Plant Physiol. 150, 889–903. 10.1104/pp.109.138677 19376835 PMC2689975

[B41] Schmidt M. Gessner G. Luff M. Heiland I. Wagner V. Kaminski M.. (2006). Proteomic analysis of the eyespot of *Chlamydomonas reinhardtii* provides novel insights into its components and tactic movements. Plant Cell 18, 1908–1930. 10.1105/tpc.106.041749 16798888 PMC1533972

[B42] Schröter Y. Steiner S. Matthai K. Pfannschmidt T. (2010). Analysis of oligomeric protein complexes in the chloroplast sub-proteome of nucleic acid-binding proteins from mustard reveals potential redox regulators of plastid gene expression. Proteomics 10, 2191–2204. 10.1002/pmic.200900678 20340159

[B43] Soll J. Schleiff E. (2004). Protein import into chloroplasts. Nat. Rev. Mol. Cell Biol. 5, 198–208. 10.1038/nrm1333 14991000

[B44] Stauber E. J. Fink A. Markert C. Kruse O. Johanningmeier U. Hippler M. (2003). Proteomics of *Chlamydomonas reinhardtii* light-harvesting proteins. Eukaryot. Cell 2, 978–994. 10.1128/EC.2.5.978-994.2003 14555480 PMC219354

[B44a] Steiner S. Dietzel L. Schröter Y. Fey V. Wagner R. Pfannschmidt T. (2009). The role of phosphorylation in redox regulation of photosynthesis genes psaA and psbA during photosynthetic acclimation of mustard. Mol. Plant 2, 416–429. 10.1093/mp/ssp007 19825626

[B45] Steiner S. Schröter Y. Pfalz J. Pfannschmidt T. (2011). Identification of essential subunits in the plastid-encoded RNA polymerase complex reveals building blocks for proper plastid development. Plant Physiol. 157, 1–13. 10.1104/pp.111.184515 21949211 PMC3252157

[B46] Stoebe B. Maier U. G. (2002). One, two, three: nature's tool box for building plastids. Protoplasma 219, 123–130. 10.1007/s007090200013 12099212

[B47] Su P. H. Li H. M. (2008). Arabidopsis stromal 70-kD heat shock proteins are essential for plant development and important for thermotolerance of germinating seeds. Plant Physiol. 146, 1231–1241. 10.1104/pp.107.114496 18192441 PMC2259073

[B48] Sugiura M. (1992). The chloroplast genome. Plant Mol. Biol. 19, 149–168. 10.1007/BF00015612 1600166

[B49] Sun Q. Zybailov B. Majeran W. Friso G. Olinares P. D. B. Van Wijk K. J. (2009). PPDB, the plant proteomics database at cornell. Nucleic Acids Res. 37, D969–D974. 10.1093/nar/gkn654 18832363 PMC2686560

[B49a] Suzuki J. Y. Ytterberg A. J. Beardslee T. A. Allison L. A. Wijk K. J. Maliga P. (2004). Affinity purification of the tobacco plastid RNA polymerase and *in vitro* reconstitution of the holoenzyme. Plant J. 40, 164–172. 10.1111/j.1365-313X.2004.02195.x 15361150

[B50] Thimm O. Blasing O. Gibon Y. Nagel A. Meyer S. Kruger P.. (2004). MAPMAN: a user-driven tool to display genomics data sets onto diagrams of metabolic pathways and other biological processes. Plant J. 37, 914–939. 10.1111/j.1365-313X.2004.02016.x 14996223

[B51] Tiller K. Eisermann A. Link G. (1991). The chloroplast transcription apparatus from mustard (*Sinapis alba L*.). Evidence for three different transcription factors which resemble bacterial sigma factors. Eur. J. Biochem. 198, 93–99. 10.1111/j.1432-1033.1991.tb15990.x 2040293

[B52] Tiller K. Link G. (1993). Phosphorylation and dephosphorylation affect functional-characteristics of chloroplast and etioplast transcription systems from mustard (*Sinapis-alba L*.). EMBO J. 12, 1745–1753. 8491168 10.1002/j.1460-2075.1993.tb05822.x PMC413393

[B53] Tkaczuk K. L. Dunin-Horkawicz S. Purta E. Bujnicki J. M. (2007). Structural and evolutionary bioinformatics of the SPOUT superfamily of methyltransferases. BMC Bioinformatics 8:73. 10.1186/1471-2105-8-73 17338813 PMC1829167

[B54] van Wijk K. J. Baginsky S. (2011). Update on plastid proteomics in higher plants; current state and future goals. Plant Physiol. 155, 1578–1588. 10.1104/pp.111.172932 21350036 PMC3091083

[B55] Veith T. Brauns J. Weisheit W. Mittag M. Büchel C. (2009). Identification of a specific fucoxanthin-chlorophyll protein in the light harvesting complex of photosystem I in the diatom *Cyclotella meneghiniana*. Biochim. Biophys. Acta 1787, 905–912. 10.1016/j.bbabio.2009.04.006 19397889

[B56] Wagner R. Pfannschmidt T. (2006). Eukaryotic transcription factors in plastids – bioinformatic assessment and implications for the evolution of gene expression machineries in plants. Gene 381, 62–70. 10.1016/j.gene.2006.06.022 16934950

[B57] Wiedmann B. Sakai H. Davis T. A. Wiedmann M. (1994). A protein complex required for signal-sequence-specific sorting and translocation. Nature 370, 434–440. 10.1038/370434a0 8047162

[B58] Xia C. Wang Y. J. Li W. Q. Chen Y. R. Deng Y. Zhang X. Q.. (2010). The *Arabidopsis eukaryotic* translation initiation factor 3, subunit F (AteIF3f), is required for pollen germination and embryogenesis. Plant J. 63, 189–202. 10.1111/j.1365-313X.2010.04237.x 20444226 PMC7190160

[B59] Yamaguchi K. Subramanian A. R. (2000). The plastid ribosomal proteins. Identification of all the proteins in the 50 S subunit of an organelle ribosome (chloroplast). J. Biol. Chem. 275, 28466–28482. 10.1074/jbc.M005012200 10874046

[B60] Yamaguchi K. von Knoblauch K. Subramanian A. R. (2000). The plastid ribosomal proteins. Identification of all the proteins in the 30 S subunit of an organelle ribosome (chloroplast). J. Biol. Chem. 275, 28455–28465. 10.1074/jbc.M004350200 10874039

[B61] Yang J. J. Schuster G. Stern D. B. (1996). CSP41, a sequence-specific chloroplast mRNA binding protein, is an endoribonuclease. Plant Cell 8, 1409–1420. 10.1105/tpc.8.8.1409 8776902 PMC161263

[B62] Yang J. J. Stern D. B. (1997). The spinach chloroplast endoribonuclease CSP41 cleaves the 3′-untranslated region of petD mRNA primarily within its terminal stem-loop structure. J. Biol. Chem. 272, 12874–12880. 10.1074/jbc.272.19.12874 9139750

[B63] Zhang L. Kato Y. Otters S. Vothknecht U. C. Sakamoto W. (2012). Essential role of VIPP1 in chloroplast envelope maintenance in Arabidopsis. Plant Cell 24, 3695–3707. 10.1105/tpc.112.103606 23001039 PMC3480296

