# Supplementary figures and images for: A purification strategy for analysis of the DNA/RNA-associated sub-proteome from chloroplasts of mustard cotyledons
Source: Front Plant Sci. 2014 Oct 29;5:557. doi: 10.3389/fpls.2014.00557 (PMC4212876; doi:10.3389/fpls.2014.00557)

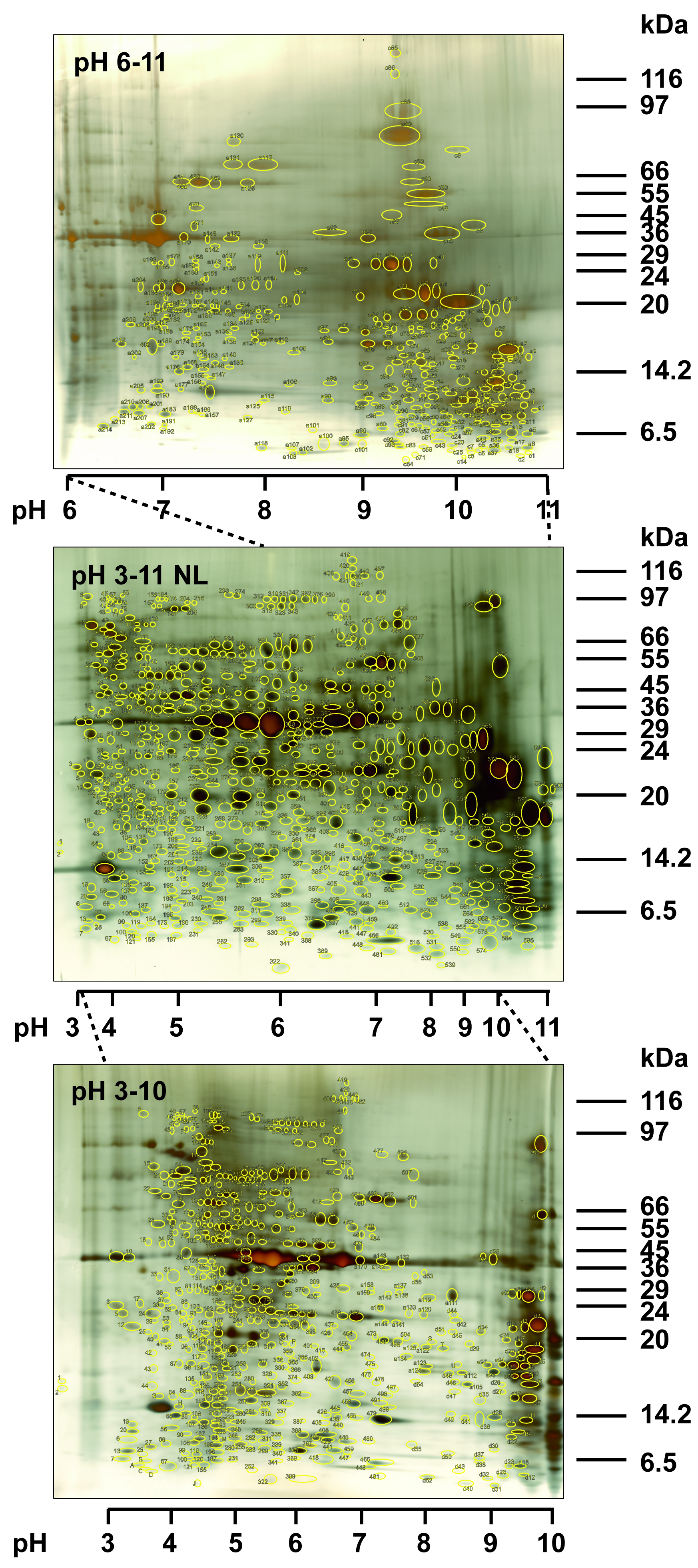

Supplement: Supplemental Figure S1 — Silver stained 2D-gels of the PC fractions with isoelectric focusing for the first dimension in pH gradients between 6–11, 3–11 NL, and 3–10 indicated in the upper left corner of each gel. The second dimension is performed in a 7.5–20% SDS polyacrylamide gel. Spots are marked and numbered in yellow. Marker sizes and pH range are given right beside and below the gel, respectively. [file Image1.JPEG]
